# Management and Outcomes of Spontaneous Cerebrospinal Fluid Otorrhoea

**DOI:** 10.3389/fsurg.2020.00021

**Published:** 2020-04-21

**Authors:** Hans GXM Thomeer, Corine Schreurs, Tristan PC van Doormaal, Louise V. Straatman

**Affiliations:** ^1^Brain Center Rudolf Magnus, University Medical Center Utrecht, Utrecht, Netherlands; ^2^Department of Otorhinolaryngology, Head and Neck Surgery, University Medical Center Utrecht, Utrecht, Netherlands; ^3^Department of Neurology and Neurosurgery, University Medical Center Utrecht, Utrecht, Netherlands

**Keywords:** skull base, CSF (cerebrospinal fluid), meningitis, otorrhoea, hearing loss conductive, surgical reconstruction primary

## Abstract

**Objective:** A cohort of patients with spontaneous cerebrospinal fluid (sCSF) otorrhoea. To report surgical outcome and discuss a treatment protocol.

**Materials and Methods:** Between 2012 and 2018 all patients presenting with sCSF were collected and data assessment was performed including clinical symptoms (hearing loss, aural fullness, meningitis, recurrent otitis media), preoperative audiometry, CT and MRI scanning. According to the site and size of the dural defect, different surgical approaches were applied.

**Results:** A total of 12 patients (14 operations) were included. Four of these had a history of meningitis. All beta-trace protein testings were positive. These patients were treated with different surgical approaches: middle fossa approach (MCF, seven patients), transmastoid approach (TMA) with bony obliteration of the cavity (three patients), and four patients underwent a subtotal petrosectomy (STP) procedure. Three cases underwent revision surgery (MCF or STP) due to residual disease (CFS leakage). After follow up duration of 13 months (6.5 months SD), no recurrence was observed. No severe adverse events such as cerebrovascular injury, meningitis, wound infection, or headache was observed in the postoperative course.

**Conclusion:** Spontaneous aural cerebrospinal fluid leakage is a rare but manageable pathology with potential severe complications. Appropriate diagnosis, laboratory testing, and imaging is primordial to obtain optimal patient outcome.

## Introduction

Cerebrospinal fluid (CSF) leakage through the lateral skull base is a rare clinical phenomenon, which can either be acquired, congenital, or spontaneous (1, 2). Acquired CSF leakage is mainly caused by skull trauma, chronic otitis media, or as a complication of previous surgery (3–5). Congenital CSF leakage can be due to tegmen defects, arachnoid granulations, inner ear malformations (such as incomplete partition or an enlarged vestibular aquaduct), or a persisting Hyrtl's fissure (6, 7).

Spontaneous CSF otorrhoea occurs in absence of all etiologies described above (8). Over the last decade the incidence of spontaneous CSF otorrhoea has nearly doubled, parallel with an increase of patients with obesity. Spontaneous CSF might be associated with idiopathic intracranial hypertension (IIH) (9–11). The chronically elevated intracranial pressure might result in thinning of the calvarial (tegmental) bone and weakening of underlying dura mater, eventually leading to permeable layers (CSF leakage).

CSF leakage might amongst others occur when a subsite of middle cranial fossa bony plate is defect (tegmen tympani, tegmen mastoideum), overlying dura becomes fragile and breaks. This defect might lead to life threatening events (such as meningitis, intracranial abscess, cerebritis), since it allows microbes to communicate with the subarachnoid space (4, 8). The risk of developing meningitis varies by etiology of CSF leakage (12). For spontaneous CSF leakage the reported rate of meningitis is around 20% (range 6–58%) (8, 13, 14), therefore its timely diagnosis is of great importance. The elapsing time before development of intracranial complications (i.e., meningitis) is still unknown. Grinblat et al. reported on a large case series (262 cases) a mean “doctor's delay” of around 34 months in spontaneous (oto-)liquorroe patients (15).

Diagnosis of otogenous CSF leakage can be difficult since it might mimic serous otitis media, with concomitant conductive hearing loss (16). Clear, pulsatile otorrhoea is often evident after myringotomy and grommet placement (5, 17). Patients can also report other non-specific symptoms such as aural fullness, tinnitus, and a headache (1). Diagnosis is confirmed by β2-transferrin or β-trace protein analysis in the fluid retracted from the ear (5, 18, 19); radiologic imaging before reconstructive surgery is primordial to inform the surgeon about the site, size, and consistency of the defect(s).

Uniformity in treatment strategy is internationally accepted, namely surgical reconstruction, however there is still a debate on the surgical approach [i.e., transmastoid (TM), middle cranial fossa (MCF) or a combination of these approaches] (20).

The objective of this study is to report on the treatment results in 13 patients with spontaneous CSF otorrhoea and to provide a diagnostic and therapeutic algorithm for decision-making in the management of CSF otorrhoea.

## Materials and Methods

### Patient Selection

A retrospective review was conducted on all patients with persistent otorrhoea, who underwent screening for CSF leakage between January 2012 and February 2018 at the University Medical Center Utrecht the Netherlands. The institutional review board of this tertiary referral center approved this study.

All adult patients clinically suspected of having CSF otorrhoea, confirmed with positive β-trace protein testing or with visible defects on radiographic imaging were included. Patients with an iatrogenic cause of CSF otorrhoea (i.e., dural lesion during otologic surgery) or posttraumatic otoliquorrhoea (after head trauma or injury) were excluded. Patients screened for nasal liquorrhoea (including anterior skull base defects) were excluded for analysis as well.

### Data Collection

Clinical records of all included patients were reviewed for demographic data, medical history, comorbidities such as idiopathic intracranial hypertension (IIH) and meningitis, medication use, otologic symptoms at presentation, β-trace protein testing, radiographic imaging, etiology of CSF otorrhoea, surgical approach, laterality, postoperative Medium care Unit stay, postoperative complications and treatment, length of follow up. Operative reports were reviewed for surgical procedure, surgical material, duration of surgery, intraoperative findings regarding the defect (location and size), perioperative application of extra cranial lumbar drainage (ELD) with intracranial pressure measurement (in cm H_2_O).

### Diagnostic Testing

β-trace protein (βTP) testing was performed on fluid obtained from the affected ear and compared to the serum levels βTP, in line with the diagnostic algorithm of Bernasconi et al. (18) The doctor's delay was defined as the time between the first presenting features of CSF leakage (chronic otorrhoea or meningitis) and the confirmed diagnosis of CSF leakage (positive βTP). Radiographic imaging consisted of multi-detector computed tomography of the temporal bone and magnetic resonance imaging. Radiographic imaging was obtained in all patients before surgery to identify the site of the defect in order to plan the surgical approach. IIH screening was assessed either by peroperative ELD measurement (drain application preoperatively to release pressure during dural elevation) or by scoring typical radiographic imaging properties (i.e., buckling optic nerve on T2 weighted MRI image, empty sella sign, hydrocephalus) or clinical assessment by a neurologist (i.e., papilledema, headache).

### Audiometric Data

The audiometric data [speech perception, pure tone average (PTA)] were obtained from audiometric test battery performed both prior and following surgery. Pure tone averages (PTA) were calculated from both air (PTA-a) and bone (PTA-b) conduction thresholds at frequencies 500, 1,000, 2,000, and 4,000 Hz (1). For these frequencies, the air-bone gap (ABG) was calculated. Word recognition score (in % correctly identified phonemes) was measured using a validated list with a minimum of 50 words with a standardized and validated format (dB) in the patient's native language.

### Surgery

Surgeries performed in this study group included middle fossa approach, transmastoid approach, with or without a bony obliteration technique, and subtotal petrosectomy.

**Figure 1:**
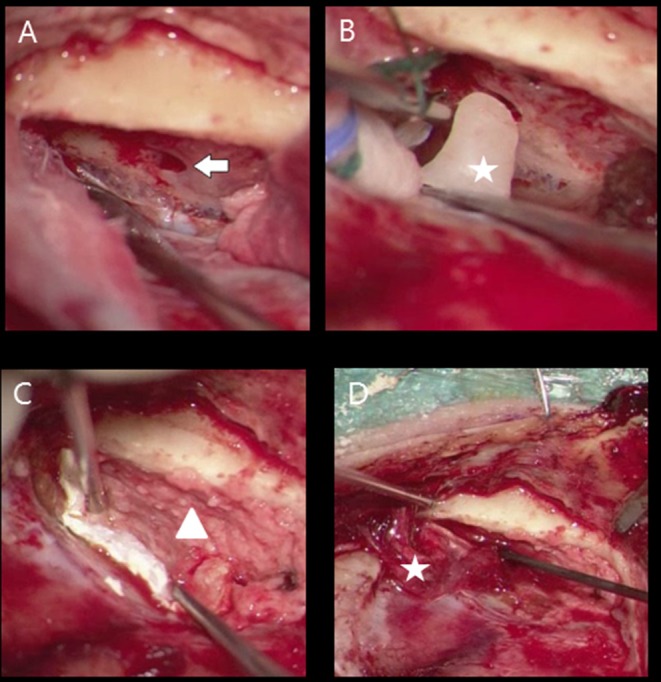
Middle cranial fossa approach meningocoele right ear **(A)** defect tegmen tympani (flash), **(B)** bone wax obliteration bony defect (star), **(C)** coverage bony tegmen surface with bone powder (triangle) and dural defect with tachosil, **(D)** fascia (star) application between bony layer (bone dust) and tachosil (dura coverage).

The middle fossa approach consisted of a supra-auricular incision centered above the bony ear canal, starting from the endaural intercartilaginous incision. An inferior pedicled temporalis fascia flap (length around 5 cm) is raised and preserved under the retractor. Vertical incision in the temporalis muscle, retractor and 2 by 3 cm bone flap (preservation of bone dust), centered superior to the bony ear canal. Then a maximum of 40 cc liquor is drained thereby lowering the dural pressure. Then identification of tegmen defect (with minimal retraction of the middle fossa dura) and subsequently application of bone dust on the defects. Then coverage of dural defect with Tachosil® (allogenous dura patch), a harvested pedicled fascia flap over the defect and usage of Tisseel (fibrine glue). Closure in three layers (Figure 1). At the end of the procedure the ELD was removed and all patients are post-operatively observed for 12 h at the postoperative medium care unit.

The transmastoid approach comprises a regular retroauricular approach with a standard mastoidectomy atticoantrotomy and identification of the dural bony defect. Either with autologous material (bone dust or chips, cartilage, fascia or a combination) or allogenous material [hydroxy appetite granules, bioactive glass (Bonalive®), bone cement (Otomimix®), Lyodura, or a combination of these] the defect is closed. In some cases the surgeons choses a bony obliteration technique (obliteration of the mastoid cavity with autogenous bone or allogenous material); this ensures defect closure depending on its location in the temporal bone. Wound closure in three layers and head bandage 24 h postoperatively.

The subtotal petrosectomy approach entails a blind sac closure of the ear canal (in two layers), removal of all squamous epithelium with tympanic membrane and ossicular chain (except the mobile stapes head and with preservation of the chorda tympani). Mastoidectomy, canal wall down procedure and closure of the tuba eustachii orifice with different materials (pedicled tensor tympani muscle, bone wax, fascia, or fat graft). Obliteration of the mastoid cavity with abdominal fat graft. Wound closure in two layers and head bandage for 48 h.

### Outcome

The primary outcome was absence of the CSF otorrhoea. Secondary outcomes were need for revision surgery, postoperative meningitis, pre- and postoperative audiometric results. Other complications were noted, such as wound infections or abscesses, wound dehiscences, postoperative bleeding, facial nerve injury, and intracranial complications (i.e., subdural hematoma, epilepsy, intracerebral ischemia).

### Statistics

A standard descriptive method for summarizing our data is conducted. Frequencies and percentages were used for nominal variables. For continuous variables, means with standard deviations or medians with ranges were used.

## Results

### Clinical Evaluation/Patient Population/Patient Description

Between 2012 and 2018, 16 patients with CSF otorrhoea were reviewed. Two patients were excluded, since they developed CSF otorrhoea after otologic surgery (mastoidectomy, ear amputation, and subtotal petrosectomy). One patient was excluded because of development of a cerebrospinal fistula after traumatic lateral skull base fracture. A total of 13 patients (13 ears) were included in this study (for details see Supplementary Table 1). Of these, 12 patients were surgically managed.

Their mean age was 59.1 years (SD 16.5 years, range 39–94). Six were male. Of all patients, 5 had undergone previous otologic surgery (atticoantrostomy, canal wall down mastoidectomy, tympanoplasty, placement of bone conduction device). Four patients had a history of chronic and recurrent otitis media with effusion. Two patients had a previous episode of CSF otorrhoea, one confirmed with BTPtesting. In both patients, CSF otorrhoea dissolved spontaneously.

In four patients, we noted a history of meningitis (case 2, 3, 4, and 12). One patient had meningitis as an intracranial complication of acute otitis media. Two patients had multiple episodes of meningitis (case 3 and 4). Two patients had severe complications of meningitis, such as intra-cerebral abscesses and sinus thrombosis (case 3 and 12).

Nine patients (69.2%) reported both symptoms of clear (pulsatile) otorrhoea and (subjective) hearing loss. Five patients (38.5%) reported otalgia ipsilateral to the CSF leak. All other symptoms and detailed description see Supplementary Table 1.

In our study, most of the CSF leaks occurred spontaneously (69.2%). Two CSF leaks were diagnosed with a concomitant infection; mastoiditis and acute otitis media. One CSF leak was due to a congenital infra-labyrinthine fissure, also known as a Hyrtl's fissure (case 8).

At presentation refractory otorrhoea and hearing loss were predominant symptoms. Therapy resistance to local and systemic antibiotics and otologic surgery without resolution of symptoms were reasons for referral at our hospital. The median delay between onset of symptoms and diagnosis (doctor's delay) was 5.3 months (IQR: 1.9–36).

### Diagnostic Testing and Imaging Studies

BTPtesting samples, either obtained from an earwick (ivalon®) or by transmembranous middle ear fluid aspiration, were positive in 12 patients (Supplementary Table 2 diagnostic testing). In two patients (case 1 and 3), two samples were needed to prove CSF otorrhoea. BTPconcentration in the ear fluid varied from 2.1 to 54 mg/L. In one patient, no BTPtesting was performed (case 12).

Radiographic imaging was performed preoperatively in all patients. In five patients cortical defects of the middle cranial fossa bony plate were confirmed, one with a tegmen tympani defect (Supplementary Table 2). In one patient the tegmen mastoideum was dehiscent with the presence of a meningo-encephalocele (case 10).

Seven patients were screened for idiopathic intracranial hypertension, three during the operation (cases 4, 7, and 11). In five patients, IIH screening was negative, in the other two patients IIH suspicion on MRI imaging (i.e., empty sella sign or kinking optic nerve) could not be confirmed with intraoperatieve testing (ELD opening pressure measurement). In 10 patients BMI could be calculated varying between 17.5 and 35 kg/m^2^ (mean 28.5, SD 5.2). Postoperative audiometric outcome could be divided in three different groups along the surgical approach (MFA, TMA, or STP). Only in the first two approaches (MFA and TMA) air conduction hearing could be preserved or improved: mean air conduction gain of 18 dB (15–20 dB range). In the subtotal petrosectomy approach no conductive hearing could be preserved.

### Surgical Approach

**Figure 2:**
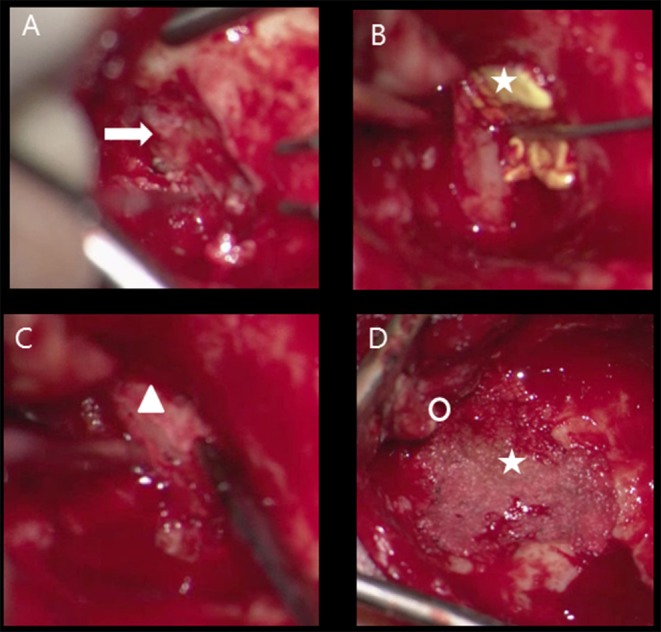
Transmastoid approach meningocoele left ear, obliteration mastoid cavity **(A)** defect tegmen mastoideum (flash), **(B)** tachosil coverage dural defect (star), **(C)** reconstruction bony tegmen defect with bone chip (triangle) and dural defect with tachosil, **(D)** obliteration cavity with bone dust (star), posterior from new bony ear canal (circle).

Seven patients underwent a MFA, all received placement of a lumbar drain just before start of surgery (Supplementary Table 3 Surgical characteristics). No other anesthetical precautions (such as arterial lining, anti-epileptic drugs) during or direct after surgery were necessary. Three patients underwent TMA (case number 3, 6, 7). In case number 3 and 6, bony obliteration of the mastoid cavity (autologous material) was performed (Figure 2). During postoperative follow up residual disease appeared in case numbers 6 and 7. In both cases, MFA was performed as salvage surgery after recurrence of CSF otorrhoea.

Four patients underwent STP; one out of these four cases received a lumbar drain for 6 days preoperatively because of a recurrent CSF leak after MFA (case number 2).

The patient with Hyrtl's fissure (case number 8) was filled up with fascia, bonewax, and surgicell.

The mean duration of surgery was 225 min (range 178–353 min). Patients were followed up for ~12.7 months (SD 6.5 months).

### Complications and Recurrence

In our study, there were no cases of postoperative meningitis, sub- or epidural bleeding or hematoma, no postoperative facial nerve palsy, nor cerebral ischemia of the temporal lobe. No postoperative perceptive hearing loss was encountered. In case 1 retro auricular infection occurred, treated with combined local and systemic antibiotics. In case 2.2 and 8 temporary loss of taste was reported. In case 7.2 the ELD drain tip was left behind inadvertently between processus spinosus L5 and S1. Surgical tip removal was performed without any postoperative complication. Case 11 necessitated a transmastoid (TMA) plugging of the superior semicircular canal in the operated ear 13 months after the first surgery (MFA). No residual liquorroe was noticed on the long term.

Of all 15 surgeries, 3 were complicated by recurrence of CSF otorrhoea (case 2.1, 6.1, 7.1). For MFA, 1 out of 7 surgeries had a recurrence of CSF otorrhoea during a follow up of 3 weeks (case 2). In this case clinical recurrence (otorrhoea with halo's on the pillow) of CSF otorrhoea on the right ear occurred 9 days after MFA. After revision surgery (subtotal petrosectomy right ear) no postoperative CSF leak occurred.

For TMA, 2 out of 3 surgeries had a recurrence of CSF otorrhoea during a follow up of respectively 5 and 8.5 months, (cases 6.1, 7.1) Supplementary Table 1. After revision surgery (middle fossa approach) no CSF leak re-occurred.

In the STP group no revision surgery was needed.

## Discussion

In this case series 13 spontaneous otoliquorrhoe patients are outlined in detail. In general, surgical treatment is a viable option leading to highly satisfactory outcome. This is in line with other reports in the literature (13, 21, 22). Moreover, the reason for surgery in most cases was a chronic, therapy resistant running ear (aural fullness, hearing loss, otitis). The minority of patients presented after a meningitis due to otogenic focus (23%). It is valuable to investigate this group of patients to improve patient counseling, prevent severe complications (meningitis, brain abcess, death) and achieve optimal treatment outcome.

In this study group the period between patient complaints and diagnosis varies between 2 weeks and 40 years with a median of 5.3 months.

Spontaneous otogenous liquorrhoe is a rare pathology, mimicking serous otitis media or chronic ear disease (refractory running ear with a bacterial of fungal infection that is often resistant to antibiotic and surgical therapy due to chronic liquor exposure). Therefore, the clinician might easily miss the diagnosis. Especially if after local/systemic treatment (nasal decongestant, tympanic membrane paracentesis, antibiotics), complaints persist. Most often, due to the resulting mucosal congestion by chronic CSF exposure, a thin tegmental (tympanic or mastoidal) defect closes off and leads to a temporary dry ear consequently prolongates the time to diagnosis; in some cases (with positive BTPtesting) active surgical treatment is postponed until the moment of recurrent disease (case 5). Especially, in isolated meningoceles (not meningoencefaloceles) this clinicopathologic pattern is seen and seems to deceive the clinician for months (sometimes years). Patients with lateral skull base defects leading to a secondary rhinorrhea were excluded from this study to obtain a cohort as a homogenous as possible.

One of the supported underlying pathophyiologic mechanisms for developing spontaneous dural defects with leakage of cerebrospinal fluid, is presence of an idiopathic intracranial hypertension (IIH). Due to this chronic increased intracranial pressure (ICP) the skull base bone density might decrease and finally disrupt. It is believed that increasing amount of obese patients correlates with the increasing number of patients diagnosed with spontaneous CSF leakage (2). Although the exact underlying mechanism of this condition is still poorly understood, there are several theories about the relationship between increased ICP and obesity (23). First of all it is hypothesized that the intracranial pressure increases as a result of intracranial venous hypertension and/or increased cerebrospinal fluid outflow resistance caused by obesity (15). In addition, Rabbani et al. recently demonstrated a strong correlation between obstructive sleep apnea (OSA) and skull base thinning (24). It has been shown that during sleep in untreated OSA patients (apneic periods), intracranial pressure frequently rises (spiking shown on polysomnographic investigation). This in contrast with isolated obesity or overweight: in these patients no strong correlation with spontaneous CSF leakage was found (9). In our patient cohort no patient was known to have elevated ICP or OSA. Nevertheless, in four out of 10 measured cases, BMI was >30 (case 2, 4, 6, and 13), showing 25% of obesity in this patient cohort. As ICP measurement is not standard in our Department during surgery and not all patients necessitated lumbar drainage (most STP or TMA cases), only in three patients opening ICP could be assessed: 17, 14, and 16 cm H_2_O in cases 4, 7, and 11 respectively (Supplementary Table 2).

Secondly, a cause for spontaneous CSF leakage might be chronic or recurrent otitis media and inflammations in the temporal bone, thereby obstructing proper development of cortical bone during infancy/adulthood (13). In these cases bilateral disease and thus thinning of the middle fossa on both sides in the same patient is suspected. In our series (13 patients), only in two patients bilateral indication for surgery is planned to reconstruct the bony skull base.

In the presented group of patients three different surgical strategies are followed; in three patients revision was needed due to recurrence of CSF leakage and another approach was employed (cases 2, 6, 7; Supplementary Table 3). Though the type of approach for surgical closure of the defect depends merely on the location of the defect, a different technique might be chosen for another reason. In case 2 for example, the indication for surgery was history of otogenic meningitis. After unsuccessful MFA attempt of reconstruction, subtotal petrosectomy approach has been applied. The latter might be seen as the ultimate option, in all cases resulting in total postoperative conductive hearing impairment. Therefore, in these cases (like in case 1), a bone conduction hearing device (Type Cochlear Baha® or Oticon BAHS®) is positioned to revalidate, this unilateral conductive hearing impairment.

Regarding the surgical approach to be chosen, whatever the approach, the philosophy to reach maximal structure and function preservation (hearing) seems preferable without recurrence of disease. Therefore, in our experience, patients with epitympanic skull base defects and an intact ossicular chain should be surgically treated via a MFA with preservation of the middle ear and ossicular chain, especially if there is limited space between the tegmen and the ossicular chain. We recommend this technique in these cases over a transmastoid approach as we had two cases (case 6 and 7) where there was a persistent epitympanic defect, just anterior to the reconstruction after a transmastoid approach. Revision surgery via MFA was applied, resulting in stable and dry ears after 9 months of follow up. A transmastoid revision surgical procedure would request an ossicular chain disruption (incus and malleushead resection) to adequately reach the defect, resulting in conductive hearing loss. The risks of any surgery need to be discussed with the patient; although we did not have any intracranial complication after MFA, there is a potential risk of meningitis, subdural hematoma and seizures after MFA that needs to be discussed with the patient (4, 8). During radiographic work up rather often a thin (or absent) bony covering of the superior canal is encountered (Supplementary Table 2): only in one case revision surgery with transmastoid (TMA) plugging of the superior semicircular canal after MFA closure of the dural defect was necessary (case 11). If the defect is merely located in the mastoid (posterior fossa or tegmen mastoideum) a transmastoid approach is indicated with reconstruction of the tegmen defect, with or without a bony obliteration of the mastoid. Nowadays, the bony obliteration technique, first developed in the eighties of the last century by Ulf Mercke (25), seems to gain more and more interest in the otologic world regarding treatment of chronic otomastoiditis and cholesteatoma cases (25–27). With upcoming high definition imaging techniques (non-echo planar diffusion weighted MRI scan) this might be regarded as a save and reliable technique, especially in cases with liquorroe risking otogenous meningitis. A subtotal petrosectomy is chosen (excluding the mastoid-middle ear cavity with obliteration of tubal orifice and cavity with abdominal fat) when no useful hearing is present preoperatively or when recurrence of liquorroe is observed; this was the case in patient 1 and 2, respectively (see Supplementary Table 3). Importantly, patients can only be treated according to this strategy **(**see treatment algorithm in Supplementary Table 4, if the available surgical team has experience in the different approaches mentioned. Otherwise, referral to specialized centers might be advisable to be able to provide optimal treatment.

Another important part of the surgery is the type of reconstruction of the dural and bony defect. Overall, consensus is reached regarding the need for multilayer reconstruction to reach maximal success rates (13, 21, 22). However, no uniformity can be obtained when the type of material is concerned: autologous (bony chips, bone dust, cartilage, fascia temporalis, tensor fascia lata, muscle) vs. allogenous (lyodura®, otomimix®, Tutoplast® fascia, Tachosil®, bone wax®, nylon dural suture) materials are applied or a combination of these. In our experience, a 4 layer combined reconstruction in MFA leads to robust and successful closure after maximum follow up (6–18 months): tachosil to cover the dural defect, a bony chip (reconstruction of the bony defect) with overlying bone dust and fascia temporalis with fibrin glue (Tisseel®) in between these layers serves as extra stability. Sometimes extra closure of different cortical air cells are sealed off using bone wax.

During seven surgeries [patient 2 (twice), 4, 7, 10, 11, and 13] an extra cranial liquor drain (ELD) was applied during surgery. This increases visibility of the middle fossa bony plate, reduces venous bleeding significantly without need for temporal lobe retraction. At the end of surgery the ELD is removed without any long term postoperative complication in all cases. In addition, the ELD served as a diagnostic tool to evaluate the intracranial pressure. It must however be taken into account that the pressure was measured before reconstruction, therefore the test can be false negative as a result of the leakage. In our study group there was no individual that developed increased intracranial pressure after closure of the defect, based on the history. However, no post-operative pressure measurements were performed.

The weakness of this study lies in the small size of the study group and its retrospective character, therefore a bias in patient selection cannot be neglected influencing outcome. Moreover, many other patients might still be having chronic discharging ears overlooking possible recurrent spontaneous CSF leakage. Five out of the 12 surgically managed patients had previous otologic surgery, this is an important factor confounding results. However, previous otologic surgery of patient ears deemed to suffer from chronic otitis media whereas the true origin might have been CSF otorrea, supports our hypothesis and suspicion of an misdiagnosed spontaneous CSF leakage. In addition, the risk of developing meningitis (around 20%, a serious complication of spontaneous CSF leakage) seems to be based on older publications in the literature. Regarding our case series, only four of 13 patients were reported with history of meningitis (around 23%); it may be that the occurrence of meningitis in these patients, might be overrated.

Future studies should highlight the diagnostic process before treatment and especially the beta-trace protein test should be re-tested: this is an easy-to-use laboratory test with quick (within 24 h) outcome. It has a high sensitivity and specificity. We would like to investigate and recalculate prospectively the BTP outcome in this group of patients. An example of a prospective cohort study would be patient group of two arms: one with regular otitis media with effusion and the other with proven CSF otorrhoea. To test the BTP outcome between these two groups is informative and reduces the amount of false positive tested patients. Also an important aspect of this subject is the causative role for obesity. We would measure all these patients BMI and screen for obstructive sleep apnea. In our small cohort of patients this was not possible, however in a multicenter prospective cohort study this might provide useful information regarding understanding and optimal treatment management of this group of patients.

## Conclusion

Spontaneous cerebrospinal fluid leakage is a rare pathology which can be successful treated via surgical reconstruction. The choice of surgical approach depends on different factors: residual hearing, location of defect, patient desire, experience of the surgical team in the different approaches. Future studies will aid in further diagnostic instrumentation.

## Data Availability Statement

The datasets generated for this study are available on request to the corresponding author.

## Ethics Statement

The studies involving human participants were reviewed and approved by Medical Ethic Committee University Medical Centre Utrecht. Written informed consent for participation was not required for this study in accordance with the national legislation and the institutional requirements.

## Author Contributions

All authors listed have made a substantial, direct and intellectual contribution to the work, and approved it for publication.

## Conflict of Interest

The authors declare that the research was conducted in the absence of any commercial or financial relationships that could be construed as a potential conflict of interest.
