# Supplementary material for: Management and Outcomes of Spontaneous Cerebrospinal Fluid Otorrhoea
Source: Front Surg. 2020 Apr 21;7:21. doi: 10.3389/fsurg.2020.00021 (PMC7186757; doi:10.3389/fsurg.2020.00021)
Supplement: Supplementary file 1 [file Table_1.docx]

| **Case** | **Age** | **Gender** | **Relevant history** | **History of meningitis** | **Presenting features** | **BTP testing** | **Doctor’s delay** | **Etiology CSF leakage** | **Treatment** | **Follow up after surgery** |
| --- | --- | --- | --- | --- | --- | --- | --- | --- | --- | --- |
| 1 | 94 | male | Acute mastoiditis, AAT | No | Hearing loss AS,  Clear pulsatile  otorrhea AS | + | 4.5 months | Acute mastoiditis AS | STP AS | 15 months |
| 2 | 72 | female | ATE, grommet AD | Yes, after AOM | Clear otorrhea AD  Hearing loss AD  Otalgia AD  Fever | + | 1.5 months | Complicated AOM AD with meningitis | 1. MFA AD  revision 2. STP AD  and BCD | 1. 2 months  2. 18 months |
| 3 | 72 | male | Canal Wall Down mastoidectomy AS, revision mastoidectomy, transient CSF otorrhea AD | Yes, multiple episodes, intracerebral abscess and subdural empyema ^(B)^ | Clear otorrhea AS | + | 4 months | Spontaneous CSF otorrhea AS | TMA+ BO AS | 6 months |
| 4 | 47 | female | None | Yes, multiple episodes of meningitis | Hearing loss AD, OMA AD,  Headache,  Vomiting,  Brudzinski’s neck sign + | + | 2 weeks | Spontaneous CSF otorrhea AD with meningitis | MFA AD | 11 months |
| 5 | 82 | male | OME AS, grommet AS | No | Pulsatile, clear otorrhea AS  Hearing loss AS | + | 6 months | Spontaneous CSF otorrhea AS | None | 18 months ^(C)^ |
| 6 | 45 | female | Tympanoplasty AD, chronic OME AD, grommets AD | No | Otorrhea AD  Otalgia AD  Tinnitus AD  Hearing loss AD | + | 18 months | Spontaneous CSF otorrhea AD | 1. TMA + BO AD revision  2. MFA AD | 1. 12 months  2. 9 months |
| 7 | 52 | female | None | No | Progressive Hearing loss AD  Pulsatile Tinnitus | + | 10 months | Spontaneous CSF otorrhea AD | 1. TMA AD revision  2. MFA AD | 1. 4 months  2. 8 months |
| 8 | 43 | female | CSF otorrhea after grommet placement ^(A)^ | No | Hearing loss AD  Mild otalgia AD | + | 40 years | Hyrtl’s fissure AD | Combined Approach with closure HF, PORP AD | 12 months |
| 9 | 47 | male | Chronic otitis AS  Grommet AS | No | Hearing loss AS  Otorrhea AS  Tinnitus AS | + | 3.5 years | Spontaneous CSF otorrhea AS | STP AS | 14 months |
| 10 | 39 | female | Grommet AS | No | Pulsatile, clear otorrhea AS  Otalgia AS | + | 3 months | Spontaneous CSF otorrhea and meningoencefalocèle AS | MFA AS | 12 months |
| 11 | 77 | female | Grommet AD | No | Hearing loss AD  Tinnitus AD  Otorrhea AD  Otalgia AD  Vertigo AD | + | 4 months | Spontaneous CSF otorrhea AD | MFA AD | 15 months |
| 12 | 55 | Male | Mastoidectomy AD, grommet AD | Yes, complicated by sinus trombosis | Headache | NA | 1 month | Spontaneous CSF otorrhea AD | STP + BCD AD | 13 months |
| 13 | 50 | male | Chronic otitis AD, grommet AD, Tympanoplasty AD | No | Clear pulsatile otorrhea AD  Otalgia AD  Tinnitus AD | + | 11 years | Spontaneous CSF liquorroe AD | MFA AD | 13 months |

**Supplementary Table 1 Case characteristics**

(A): anamnestic, not confirmed by BTP testing or radiographic imaging (B): two episodes of meningitis, one complicated by intracerebral abscess and therefore temporal trepanation, also a subdural empyema therefore mastoidectomy AD. (C): follow up in another hospital

Abbreviations: AS, auris sinister; AD, auris dextra; ATE, adenotonsillectomy; AAT, atticoantrostomy; AOM, acute otitis media; STP, subtotal petrosectomy; TMA, transmastoid approach; MFA, middle fossa

Approach; BO, bony obliteration of mastoid cavity; HF, Hyrtl’s fissure; BCD, bone conductive device; TM, tympanic membrane; CAT, combined approach tympanoplasty; BCD, bone Conduction Device; NA, not applicable

Definition: Doctor’s delay, time between first presenting features of CSF leakage and confirmed CSF leakage (positive β-trace protein test or visible defect middle cranial fossa bony plate on CT or MRI)
