# Supplementary material for: Management and Outcomes of Spontaneous Cerebrospinal Fluid Otorrhoea
Source: Front Surg. 2020 Apr 21;7:21. doi: 10.3389/fsurg.2020.00021 (PMC7186757; doi:10.3389/fsurg.2020.00021)
Supplement: Supplementary file 2 [file Table_2.docx]

| **Case** | **BTP (mg/L)** | **Radiographic imaging** | **BMI (kg/m^2^)** | **IIH (ICP: cmH20)** |
| --- | --- | --- | --- | --- |
| 1 | Sample 1: 1.3  Sample 2: 1.2 | CT mastoid AS: no defect tegmen tympani.  MRI not performed | 29.7 | NA |
| 2 | Sample 1: 11 | CT mastoid AD: no bony defect skull base, no dehiscence  MRI CPA AD: bony defects in point of temporal bone and medially above sigmoid sinus, no meningoencephalocele | 32.3 | NA |
| 3 | Sample 1: 0.28  Sample 2: 3.7 | CT mastoid AS: postoperative status with large defect towards middle cranial fossa with soft tissue, meningoencephalocele.  MRI cholesteatoma / CPA: high signal mastoid AD. Pre-existing encephalocele mastoid AD | 27.4 | NA |
| 4 | Sample 1: 4.6 | CT mastoid AD: subdural/subarachnoid air in the area of sigmoid sinus, no midlineshift, no visible defect  MRI not performed | 31.6 | No (17) |
| 5 | Sample 1: 4.2 | CT mastoid AS: no tegmen defect, soft tissue in Prussak, cholesteatoma not ruled out  MRI CPA: pituary adenoma | NA | NA |
| 6 | Sample 1: 3.0  Sample 2: 6.4 | CT mastoid AD: no tegmen defect or skull base defect  MRI cerebrum: no pathologic findings | 35 | NA |
| 7 | Sample 1: 9.4  Sample 2: 7.4 | CT mastoid AD: no defect tegmen or other bony defects  MRI CPA: No signs of IIH | 17.5 | No (14) |
| 8 | Sample 1: 12 | CT mastoid AD: persisting Hyrtl’s fissure  MRI CPA: no tegmen defect. Small focus with diffusion restriction in aditus ad antrum | 22.2 | NA |
| 9 | Sample 1: 2.1 | CT mastoid AS: no tegmen defect or other bony defect  MRI CPA: no meningoencephalocele | NA | NA |
| 10 | Sample 1: 27 | CT mastoid AS: tegmendefect of 7mm with soft tissue in epitympanum  MRI CPA: herniation of soft tissue of left temporal lobe through tegmen defect | 29.3 | NA |
| 11 | Sample 1: 54 | CT CPA AD: Superior canal dehiscence, but symmetrical thin bony barrier of additus antrum  MRI CPA AD: no pathology | 27.3 | No (16) |
| 12 | NA | CT mastoid AD: poorly developed mastoid. Soft tissue in antrum. Bony barrier of roof and front of epitympanum not visible, absent.  MRA CPA: diffusion restriction mastoid and middle ear | NA | NA |
| 13 | Sample 1: 7.3 | CT temporal bone AD: tegmen defect, ventral osseous barrier of middle is not visible  MRI CPA: T2 hyper intensity around tegmen defect, possibly cerebrospinal fluid leakage | 32.5 | NA |

**Supplementary Table 2 Diagnostic testing**

Abbreviations: BTP, bèta trace protein; AS, auris sinister; AD, auris dextra; IIH, idiopathic intracranial hypertension; MRI CPA, MRI of cerebellopontine angle; NA, not applicable; BMI, body mass index (=weight/length^2^); ICP, intracranial pressure
