# Supplementary material for: Management and Outcomes of Spontaneous Cerebrospinal Fluid Otorrhoea
Source: Front Surg. 2020 Apr 21;7:21. doi: 10.3389/fsurg.2020.00021 (PMC7186757; doi:10.3389/fsurg.2020.00021)
Supplement: Supplementary file 3 [file Table_3.docx]

| **Case** | **Surgical approach** | **Surgical material** | **Duration surgery (min)** | **ELD** | **MCU (days)** | **Postoperative complications** | **Treatment *** |
| --- | --- | --- | --- | --- | --- | --- | --- |
| 1 | STP AS | Bone wax, Soft tissue, Abdominal fat, Tisseel TG | 180 | No | No | Retro auricular wound infection | Clindamycine 8 days,  Azithromycin 3 days,  ofloxacine AS 8 days,  TCT ointment 7 days |
| 2.1 | MFA AD | Nylon dural suture, temporalis muscle, tisseel, tachosil and lyostipt, bone chip | 277 | Yes, 6 days | Yes, 12h | Recurrence CSF otorrhea | Re-ELD placement  STP AD |
| 2.2 | STP AD | bone wax, tensor tympani muscle, abdominal fat | 185 | Yes, 6 days | No | None | Augmentin (7 days) |
| 3 | BO AS | Sealed lateral with bonewax and surgicel, reconstruction posterior wall with autologous bone, dural coverage with tachosil, fascia m. temporalis, TG | 222 | No | No | None | None |
| 4 | MFA AD | Autologous cortical bone(dust) on tegmen defect with overlying inferior pedicled temporalis fascia flap | 256 | Yes (IO) | Yes, 24h | None | None |
| 5 | NA | NA | NA | NA | NA | NA | NA |
| 6.1 | BO AD/TMA AD | Autologous cortical bone for obliteration tegmen defect, tachosil with fascia flap and TG | 208 | No | No | Recurrence CSF otorrhea | MFA AD |
| 6.2 | MFA AD | Bonewax, Autologous cortical bone(dust), pedicled temporalis fascia flap, Tachosil | 148 | Yes (IO) | Yes, 12h | None | None |
| 7.1 | TMA AD | Closure defect with tachosil, cartilage, autologous cortical bone(dust and span), TG, obliteration mastoid with cortical bone (dust) | 213 | No | No | Recurrence CSF otorrhea | MFA AD |
| 7.2 | MFA AD | Autologous cortical bone(span), closure dura defect with tachosil, autologous cortical bone (dust), TG, overlay fascia (m. temporalis) | 211 | Yes (IO) | Yes 24h | Corpus alienum (tip ELD drain) | Surgery to remove tip ELD drain (processus spinosus L5-S1) |
| 8 | CAT with closure HF AD | Fascia, bonewax, surgicel and tisseel with fascia in opening of the fissure | 276 | No | No | Transient taste disorder | None |
| 9 | STP AS with blind sac closure and abdominal fat obliteration | Obliteration cavity with abdominal fat and TG | 353 | No | No | No | None |
| 10 | MFA AS | Autologous cortical bone(span), tachosil, TG, autologous cortical bone(dust), bone wax | 252 | Yes (IO) | Yes, 24h | None | Profylactic augmentin 7 days, trafloxal ointment 7 days |
| 11 | MFA AD | Bonewax, tachosil, autologous cortical bone (dust), boneflap | 186 | Yes (IO) | Yes, 24h | Postoperative atrial fibrillation (mild potassium deficiency)  Vertigo (Superior canal syndrome symptoms) | Potassium supplementation, atenolol, rivaroxaban  TMA, plugging superior canal right ear |
| 12 | STP + BCD AD | Bonewax, periost, abdominal fat, fascia, closure in 3 layers | 178 | No | No | None | None |
| 13 | MFA AD | Autologous cortical bone(span, dust), tachosil, tisseel TG, pedicled fasciaflap overlay | 226 | Yes (IO) | 24h | None | None |

**Supplementary Table 3 Surgical characteristics**

*Treatment of postoperative complication, persisting CSF leak or recurrent CSF leak

Abbreviations: AS, auris sinister; AD, auris dextra; STP, subtotal petrosectomy; TMA, transmastoid approach; MFA, middle fossa approach; BO, bony obliteration of mastoid cavity; HF, Hyrtl’s fissure; CAT, combined approach tympanoplasty; OCR, ossicular chain reconstruction; BCD, bone conduction device; NA, non applicable; h, hours; TG, tissue glue; ELD, extracranial lumbar drainage; MCU, postoperative medium care unit stay; IO, Intraoperatively
