# Supplementary material for: Management and Outcomes of Spontaneous Cerebrospinal Fluid Otorrhoea
Source: Front Surg. 2020 Apr 21;7:21. doi: 10.3389/fsurg.2020.00021 (PMC7186757; doi:10.3389/fsurg.2020.00021)
Supplement: Supplementary file 4 [file Table_4.docx]

**Flow Chart Treatment CSF** **Otorrhoea**

Patient with spontaneous otorrhoea with/-out otitis media

Resolution:
- Spontaneous
- After medical treatment

No

Conservative Treatment

* In cases where space (>0.8mm) between intact ossicular chain and tegmen tympani defect: then only underlay reconstruction tegmen defect with bony mastoid obliteration, without ossicular chain sacrifice

Yes

Yes

Beta-trace protein positive

Bony mastoid and epitympanic obliteration

Subtotal
Petrosectomy

Temporal Bone scanning (CT and MRI)

Servicable hearing

No

Yes

Middle Cranial fossa Repair

Yes

No

Tegmen Tympani defect with intact Ossicular Chain

Mastoid Defect

Bony mastoid obliteration*
